# Polyrhythm Perception and Production: A Scoping Review

**DOI:** 10.1111/nyas.70330

**Published:** 2026-06-30

**Authors:** Patti Nijhuis, Cecilie Møller, Joshua S. Bamford, Jan Stupacher

**Affiliations:** ^1^ Centre of Excellence in Music, Mind, Body and Brain Department of Music, Art and Culture Studies University of Jyväskylä Jyväskylä Finland; ^2^ Center for Music in the Brain Department of Clinical Medicine Aarhus University & The Royal Academy of Music Aarhus/Aalborg Aarhus Denmark; ^3^ Centre for the Study of Social Cohesion, School of Anthropology and Museum Ethnography University of Oxford Oxford UK

**Keywords:** beat, polyrhythm, ratio, rhythmic complexity, rhythm perception, rhythm production, tempo

## Abstract

In music, rhythms can range from simple isochrony to complex polyrhythms, in which multiple nonharmonically related metrical hierarchies are superimposed (e.g., 2:3, 4:5). This scoping review synthesizes 64 studies comprising 96 experiments on polyrhythm perception and production. Over the past decades, research has shown that factors such as polyrhythm ratio, tempo, sensory modality, and musical training shape how we perceive and produce polyrhythms. Although most reviewed studies lack subjective measurements of polyrhythm complexity, behavioral tasks suggest that ratio sum (e.g., five in 2:3) and the Farey tree offer rough complexity estimates. While tempo affects which periodicity is perceived as the underlying beat, these effects are limited by the preference for binary subdivision grouping. Finally, the reviewed literature suggests that polyrhythms are more often perceived as integrated patterns than segregated streams. For future research, we recommend defining polyrhythms as the superposition of two or more nonharmonically related pulses that share a common cycle, and adding a clear description of whether these pulses are isochronous stimuli or perceived beats extracted from nonisochronous stimuli. Combining behavioral and subjective measures across different polyrhythmic contexts could clarify subjective complexity and under which circumstances polyrhythms are perceived and produced as integrated rhythms or segregated pulses.

## Graphical abstract

Findings from 64 empirical polyrhythm studies, comprising 96 experiments, indicate that these studies focus on simple ratios and auditory stimuli, and rely on relatively small sample sizes. We conclude that polyrhythmic complexity can be roughly estimated using ratio sum and Farey trees, that tempo influences beat perception within the constraints of a preference for binary subdivision grouping, and that polyrhythms are typically perceived as integrated patterns rather than segregated streams.

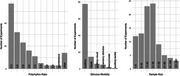

## 1 Introduction

Rhythmic patterns are fundamental to our daily lives [1, 2, 3]. We encounter them in the regularity of walking, the cadence of speech, the synchronization of social interaction, and perhaps most prominently in music. Musical rhythms can range from simple pulses, such as the four‐on‐the‐floor bass drum in some electronic music genres, to complex polyrhythms, which consist of two or more contrasting rhythmic patterns that afford multiple interpretations of the underlying beat. Polyrhythms are a fundamental aspect of musical expression found across a wide range of cultures and genres [4]; they are, for instance, prevalent in West African drumming, Indian classical music, jazz, progressive rock, European and North American modernist music, and contemporary electronic music. Given their ubiquity and cognitive complexity, polyrhythms have been a central topic in music cognition research since the late 1970s, with a peak in publications around 2010. Early research often focused on the question of whether polyrhythms are perceived as integrated or segregated perceptual entities, whereas recent studies also use polyrhythms to study individual differences, motor coordination, aesthetic judgments, and complex interpersonal interaction. Given this persistent interest and the expanding scope of research questions, a comprehensive literature review is timely for synthesizing current findings and providing recommendations for future research.

Rhythms in music are the specific patterns of notes and silences with varying durations within a musical sequence, such as the iconic temporal pattern of “Happy Birthday,” they rely on the interplay of three hierarchical levels—subdivisions, beat, and meter—each contributing uniquely to our auditory experience by creating recognizable auditory objects [4, 5, 6]. The beat is the subjective underlying pulse that guides our physical responses to music, like foot‐tapping [5, 6]. While often associated with regularly recurring note onsets, the beat is not necessarily physically present in a rhythm; it is extracted from the rhythm and exists primarily in the listener's experience [7]. In most popular music, the beat is commonly represented at the quarter note level, with subdivisions represented at the eighth note level and below (e.g., 16th, 32nd). Binary subdivisions of the beat entails grouping of two/four subdivisions while ternary subdivisions of the beat entails grouping of three subdivisions, defining what in music theory is termed simple or compound meters, respectively. Like subdivisions, beats are typically perceived in groups of two, three or four, defining what in music theory is referred to as duple, triple, or quadruple meters, respectively. Through this grouping, meter introduces a hierarchical structure to the beat, typically creating a sense of strong and weak beats within a recurring cycle [8]. It is important to note that while the notion of strong and weak beats is common in Western theoretical traditions, metrical hierarchies can take different forms depending on enculturation [4, 9]. In general, hierarchical organization is crucial for coordinating group activities like dancing, as it allows individuals to anticipate movement cues [6].

**FIGURE 1:**
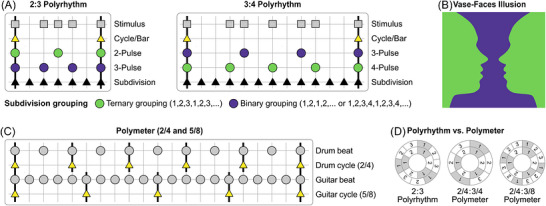
(A) Schematic representation of 2:3 and 3:4 polyrhythms (Stimulus) and their key metrical levels ranging from Subdivisions to individual Pulses to the overarching Cycle/Bar. Dark purple dots represent binary subdivision grouping (binarized pulses) and bright green dots represent ternary subdivision grouping (ternarized pulses). (B) Rubin vase as an example of an ambiguous or bistable visual form [13]. The picture can either be perceived as a dark purple vase or two bright green faces, but not as both at the same time. (C) A polymeter of 2/4 against 5/8, as featured in the song “5/4” by Gorillaz. Drum beat and guitar beat are integer multiples of each other; two guitar beats align with one drum beat. The cycle of the guitar beat (chords: C5‐C5‐F5‐F5‐F5‐C5‐C5‐Eb5‐Eb5‐Eb5) repeats every five eighth notes, while the cycle of the drum beat (kick‐snare) repeats every two quarter notes (i.e., every four eighth notes). The first downbeats of the drum and guitar cycles line up only once every 20 eighth notes; between these points, they are misaligned. (D) Schematic illustration of how polyrhythms and polymeters are defined in this study. In the 2:3 polyrhythm, the IOIs between two‐pulse and three‐pulse differ, while the cycle is shared. In the 2/4:3/4 polymeter the beat‐IOIs are shared and in the 2/4:3/8 polymeter the beat‐IOIs are integer multiples of each other, while the durations of the cycles differ.

In polyrhythms, two or more metrical hierarchies with beats that are indivisible by each other are superimposed, for example, 2:3, 3:4, 2:5, 3:5, 4:5, 5:6, 5:7, or 7:8 (see glossary in Stupacher et al. [10]). We refer to these beats as pulses of the polyrhythms. Subdivisions in polyrhythms represent the lowest common multiple of the pulse ratio. For example, a 2:3 polyrhythm has six subdivisions and a 3:4 polyrhythm has 12 subdivisions (Figure 1A). As a result, each inter‐onset interval (IOI) of the two‐pulse in a 2:3 polyrhythm spans three subdivisions, whereas each IOI of the three‐pulse spans two subdivisions. Similarly, a 3:5 polyrhythm has 15 subdivisions with the three‐pulse IOI spanning five and the five‐pulse IOI spanning three subdivisions. These subdivisions are not necessarily present in the audio signal and might not always be perceptually viable, for example, when they are too short to be perceived as individual events [11]. Listeners may perceive one or the other pulse as the metrical reference point, giving rise to two different perceptual experiences of the same polyrhythm. This affordance of multiple possible underlying beats can create a feeling of what is often described as tension or off‐balance [12]. The phenomenon is similar to the well‐known optical vase‐faces illusion [13] (Figure 1B). However, switching between perceptual interpretations in polyrhythms is often more difficult than in Rubin's bistable figure, as acoustic properties and individual differences may bias listeners toward a specific pulse [5, 10].

An important feature of polyrhythms is that the contrasting pulses share a common cycle^1^ as illustrated in Figure 1A. As a result, the IOIs of the individual pulses differ, reflecting distinct periodicities within a common cycle. In our definition of polymeters, which is typically used in music education and common practice [14], IOIs between beats in two individual meters have the same or integer multiple periodicities [15]. This means that in contrast to polyrhythms, polymeters do not share a common cycle, or bar level; each meter follows its own cycle (Figure 1C). Thus, polymeters span phrases consisting of multiple cycles [15, 16]. The difference between polyrhythms and polymeters is illustrated in Figure 1D and discussed in Section 4.1.

In recent decades, researchers across disciplines have used various terminologies to describe polyrhythms. Terms such as *cross‐rhythm* [17], *counter‐rhythm*, and even *polymeter* are sometimes used interchangeably with *polyrhythm*, despite referring to distinct concepts. At the same time, studies have referred to the 2:3 polyrhythm as an ambiguous six‐beat pattern, rather than a polyrhythm, when using it to study metrical interpretation of ambiguous rhythms [18, 19, 20, 21, 22]. There are also inconsistent views about what constitutes a simple versus a complex polyrhythm [5, 23]. One aim of the current scoping review is therefore to chart these terminologies and suggest a coherent framework for future use in rhythm perception and production research. More broadly, this review synthesizes how polyrhythms have been empirically studied—whether as a primary focus or as a methodological tool—to gain insights into human motor coordination, timing mechanisms, social interaction, and rhythm perception.

## 2 Method

### 2.1 Preregistration and Data Availability

The following method was preregistered using the Generalized Systematic Review Registration Form on OSF: https://osf.io/hr462. Data are available here: https://osf.io/9mghj/.

### 2.2 Eligibility Criteria

We included only empirical studies that investigated the perception or production of polyrhythms in human participants. No additional restrictions were applied regarding participant characteristics. Studies using polyrhythms for their perceptual ambiguity without explicitly mentioning or discussing polyrhythms were not included (e.g., 2:3 in [18, 19, 20, 24]). Gray literature, that is, work not published in a standard academic channel, was excluded.

### 2.3 Information Sources and Search Strategies

We searched the Web of Science and Scopus databases for relevant studies using the following query string applied to titles, abstracts, and keywords:

(poly‐rhythm* OR crossrhythm* OR crossbeat* OR polyrhythm* OR cross‐rhythm* OR cross‐beat* OR polymet* OR hemiola OR syncopation OR rhythm OR beat) AND (produc* OR perform* OR perceiv* OR percept* OR process* OR coordin*) AND (method* OR stimul* OR empiric* OR participant* OR results OR subject* OR paradigm OR protocol) AND (meter OR pulse OR stream* OR metric* OR hierarch* OR pattern) AND (music* OR audit* OR coordin* OR tap* OR motor).

This search strategy contains five necessary parts combined by “and” terms:

1) Synonyms and adjacent concepts of polyrhythm (poly‐rhythm* …)
2) Any form of production or perception (produc* …)
3) Indicators of empirical research to exclude purely historical, analytical, theoretical, critical, or cultural musicology (method* …)
4) Indicators of structured patterns relevant to beat and meter perception (meter …)
5) Indicators of musical, auditory, or motor rhythms to exclude biological or physiological rhythms, such as heartbeat or circadian rhythms (music* …)

No additional filters were used. The query string was validated and refined to ensure it returned specified studies listed in the preregistration: https://osf.io/hr462. The main database search was conducted in May 2024, followed by an additional search in May 2025, to include studies published in 2024 and 2025. We also used snowball sampling once the initial database was compiled. This involved searching through the reference lists of papers in the database for additional studies to include.

### 2.4 Screening

The results of the initial database search in May 2024 were imported into Zotero to merge duplicates. The merged dataset was then uploaded to ASReview, an AI‐assisted screening platform that sorts the dataset based on predicted relevance (https://asreview.nl/). Two independent screeners reviewed titles and abstracts within ASReview until 100 consecutive irrelevant records were rejected. The screening results were compared, and any discrepancies were resolved through discussion with all authors. This process was repeated in May 2025, with the exception that the ASReview stopping criterion was set to 50 consecutive irrelevant records. The databases before and after filtering are available online: https://osf.io/9mghj/.

### 2.5 Data Extraction

We extracted bibliographic information, definitions of polyrhythm, sample sizes, participant characteristics, description of methods, objectives of the studies, and implications for understanding polyrhythms. Each study was categorized as either focusing on perception or production. The data extraction sheet is available online: https://osf.io/9mghj/.

### 2.6 Quality Assessment

We extracted information about experimental blinding and sample sizes of each experiment (see *Risk of Bias* section in data extraction sheet). Because the aim of this scoping review is to provide a broad overview of polyrhythm research, we did not formally assess risks of bias. Information about the experiments’ methods was extracted for descriptive purposes rather than for study exclusion.

## 3 Results

### 3.1 Study Selection

**FIGURE 2:**
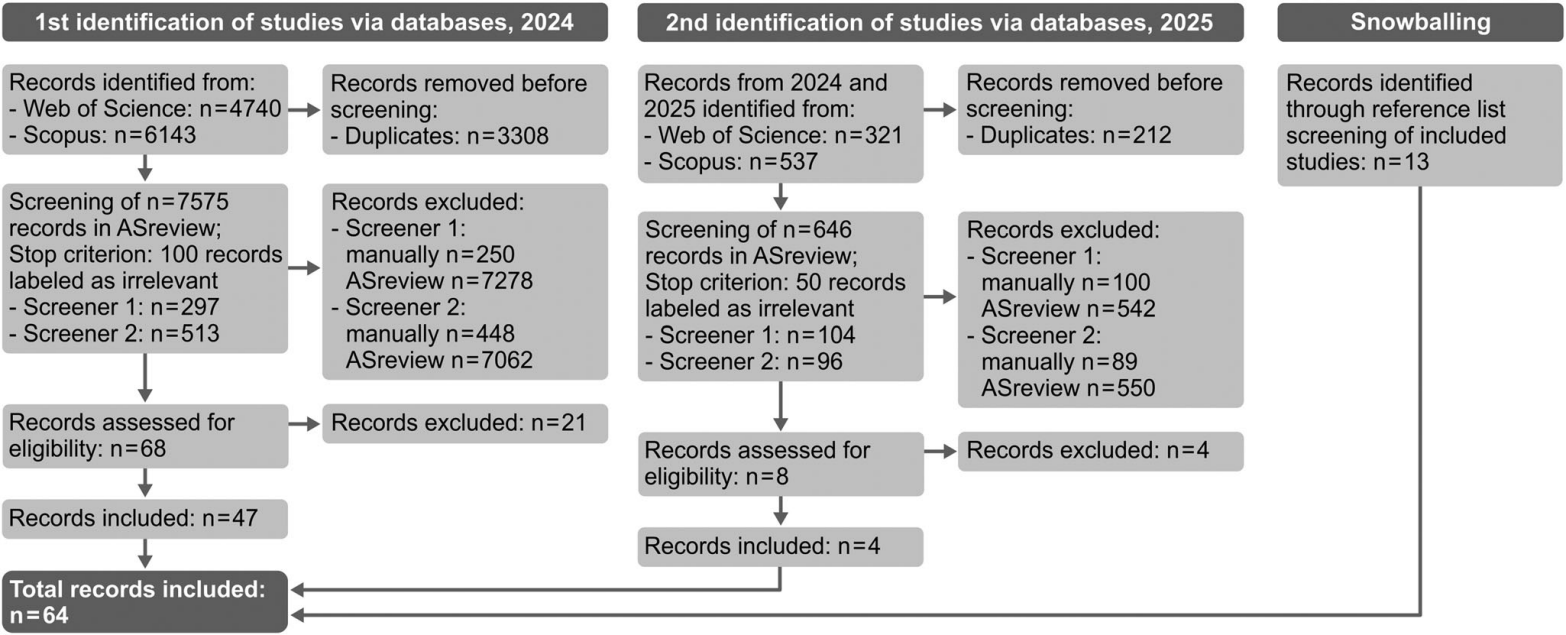
Flow chart of the study selection process.

A flow diagram of the study selection process is shown in Figure 2. In the initial search conducted in May 2024, 7575 records were identified after removing duplicates from Web of Science and Scopus. During the title and abstract screening using ASReview, both independent screeners agreed on 42 studies for inclusion. Additionally, 26 studies were suggested by only one of the screeners. The inter‐rater reliability, computed using Cohen's kappa, was 0.75, and the overall agreement was 99.6%. These statistics include all records, also the ones labeled as irrelevant by ASReview. After discussion with all the authors, one study from the set suggested by both screeners and 20 studies from the set suggested by a single screener were excluded because they were not empirical or did not study polyrhythms. The final dataset of the initial screening consisted of 47 eligible studies. In the second search conducted in May 2025, 646 records published in 2024 and 2025 were identified after removing duplicates. Three studies were suggested for inclusion by both screeners and five studies by only one screener. All three studies suggested by both screeners and one of the studies suggested by a single screener were included. Cohen's kappa was 0.54 and the overall agreement 99.2%. Thirteen additional studies were identified through snowball sampling, resulting in a final dataset of 64 eligible studies including 96 individual experiments.

### 3.2 Publication Years

**FIGURE 3:**
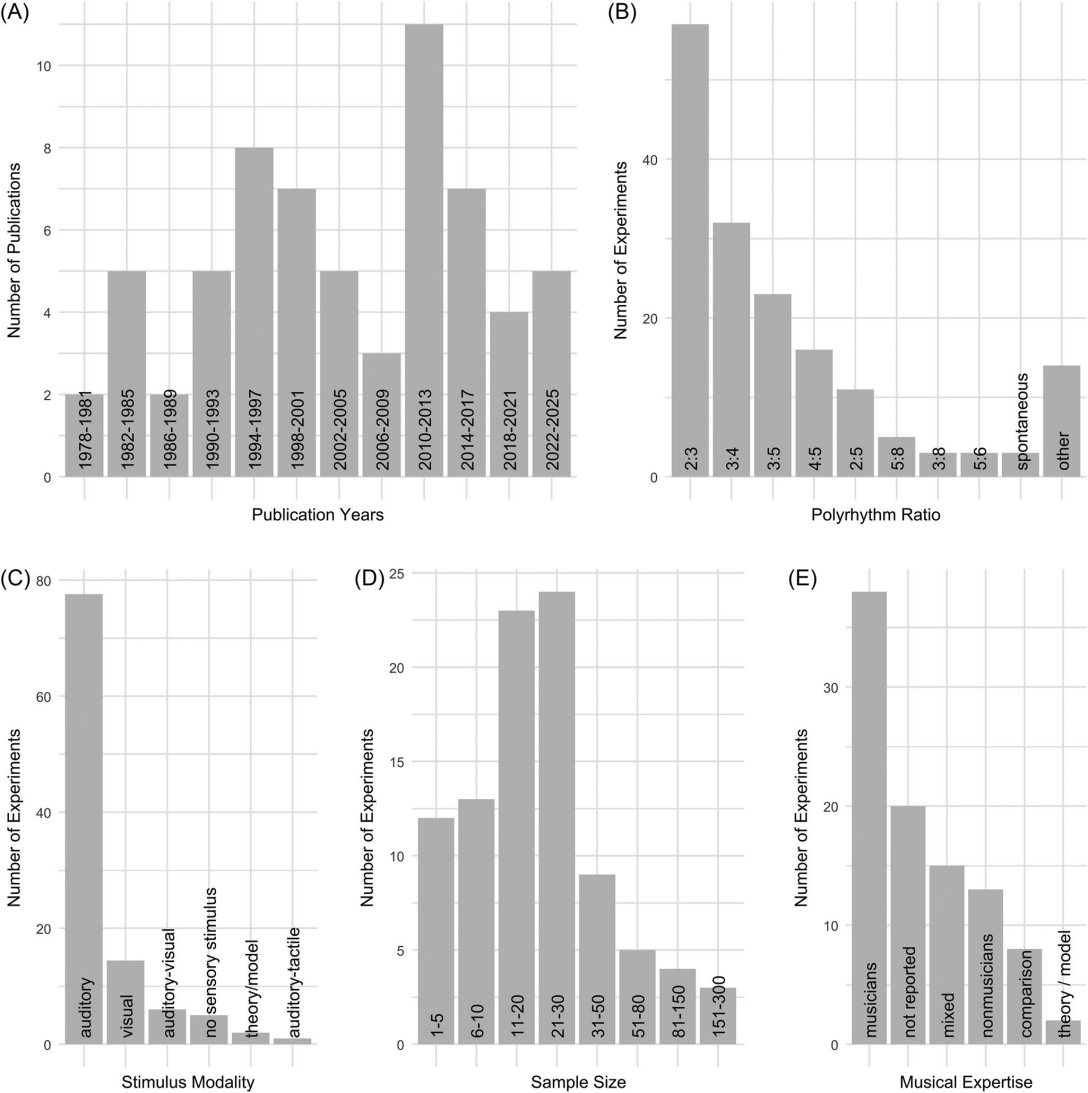
(A) Number of publications over time in 4‐year periods. (B) Number of experiments using stimuli with specific polyrhythm ratios. The “spontaneous” category includes three experiments investigating spontaneously emerging polyrhythms in bimanual coordination. The “other” category includes ratios that were used in only one or two experiments: 5:7 (*n* = 2), 3:4:5 (*n* = 2), 4:7, 7:9, 2:3:4, 2:3:7, and 2:5:7. One experiment in the “other” category used real music with various rhythms and one did not report ratios. (C) Stimulus modalities of experiments. (D) Sample sizes of experiments. (E) Musical expertise of participants in the experiments. The mixed category includes experiments with musicians and nonmusicians or a wide range of musical expertise. The comparison category includes experiments directly comparing a group of musicians with a group of nonmusicians.

The selected studies were published between 1978 and 2025 (Figure 3A). The median of publication years is 2005, indicating that half of the studies in the database were published in the last 20 years and the other half in the 27 years prior to 2005. This suggests only a slight increase in publications on polyrhythm perception and production over the years. The highest number of publications occurred between 2010 and 2013.

### 3.3 Polyrhythm Ratios

The most commonly studied polyrhythm ratio is 2:3, which was used in 56 (33.7%) individual experiments, followed by 3:4, which was used in 32 (19.3%) experiments (Figure 3B). Together, these two ratios account for more than half of the experiments, while most of the remaining experiments focus on the ratios 3:5, 4:5, 2:5, 5:8, 3:8, and 5:6. Three experiments used spontaneous movement synchronization without predefined ratios [25, 26] and one experiment used a wide range of polyrhythms in real music [27].

### 3.4 Perception Versus Production

Although the perception and production of polyrhythms are closely intertwined, we categorized the experiments based on their primary focus. Fifty‐eight experiments focused on polyrhythm production, including the following subcategories: guided bimanual, interlimb, and breathing‐movement coordination, spontaneous bimanual coordination, skill acquisition, and pattern reproduction. Thirty‐three experiments focused on polyrhythm perception, including the subcategories beat perception (measured through finger tapping), temporal deviant detection, ratings of the timing of probe tones, neurophysiological measures, pattern recognition, and passive listening. Five experiments focused on both perception and production of polyrhythms. The full list of categories is provided in the data extraction sheet: https://osf.io/9mghj/.

### 3.5 Stimulus Modalities

Seventy‐seven experiments (72.6%) presented polyrhythms auditorily (Figure 3C). Fifteen experiments (14.2%) used visual guides to present polyrhythms and six (5.7%) used a bimodal combination of auditory and visual stimuli. One experiment used a combination of auditory and tactile metronomes to guide 3:2 polyrhythmic movements [28]. Finally, five experiments (4.7%) did not provide any auditory, visual, or tactile guidance to generate bimanual polyrhythms. However, these experiments either included a familiarization phase in which polyrhythms were presented auditorily [29] or a conceptual description of polyrhythms [30, 31]. Three of these five experiments were done with musicians, one with nonmusicians, and one does not provide information about the participants’ musical expertise.

### 3.6 Participants

An overview of sample size categories is presented in Figure 3D. Twenty‐five studies had 10 or less participants, and most of these studies tested skilled musicians, limiting the generalizability of the findings (see data extraction sheet). The majority of experiments (47, 50.5%) included between 11 and 30 participants. Of the eight experiments with 80 or more participants (8.3%), data of six experiments reported in three papers were collected online [5, 10, 27]. The other two experiments (*n* = 199 and *n* = 112) used between‐subject designs with participants divided into two musical expertise groups and each group further split into five test conditions [32]. A Spearman correlation between publication year and sample size was positive, but not significant (*r* = 0.13, *p* = 0.211), indicating that sample sizes have not increased significantly over time. One study failed to report sample sizes [33] and two modeling studies did not involve participants [34, 35]. Sample sizes for all included studies can be found in the data extraction sheet: https://osf.io/9mghj/.

Thirty‐eight experiments (39.6%) exclusively tested musicians (Figure 3E). Of those 38 experiments, 27 focused on polyrhythm production. Only 13 experiments (13.5%) exclusively tested nonmusicians, and 15 (15.6%) included both musicians and nonmusicians. Eight experiments (8.3%) made a direct comparison between musicians and nonmusicians. Twenty experiments (20.8%) did not provide any information on participants’ musical expertise. Expertise in a nonmusical domain was examined by Trongjitpituk and Homma [36], who compared polyrhythm tapping accuracy in elite artistic swimmers, novice artistic swimmers, and nonartistic swimmers.

Information on dance expertise is reported in only one study, where one out of 60 participants was a regular dancer [37]. Participants’ nationality or the university where the experiment was conducted is reported in 53 of the 96 experiments (55.2%) and, when used as a proxy for cultural background, reflects predominantly Western, educated, industrialized, rich, and democratic (WEIRD) samples [38].

## 4 Discussion

### 4.1 Terminology

An often‐cited definition of polyrhythm is “the simultaneous presentation of two‐pulse trains such that the rates are not integral multiples of each other” [39]. This definition was later expanded to include the possibility of more than two‐pulse trains and to specify that each pulse train consists of a series of isochronous identical elements [17]. In both the auditory perception and bimanual rhythm production domains, the pulse trains or bimanual movement patterns of polyrhythms are frequently characterized as conflicting [17, 34, 40, 41, 42, 43, 44, 45, 46, 47], due to their inharmonic, noninteger ratios [40, 42, 45]. Earlier literature occasionally used the term *syncopated polyrhythms* to distinguish conflicting or dissonant noninteger ratios, such as 2:3 and 3:4, from integer ratios, such as 2:4 or 3:6 [17, 39, 40, 41]. More recent studies, however, define polyrhythms as noninteger or nonharmonically related ratios of pulse trains or bimanual movement patterns, thereby implicitly excluding integer or harmonically related ratios [5, 10, 23, 31, 35, 36, 45, 46, 48, 49, 50].

In slight contrast to other studies, Poudrier and Repp [23] and Poudrier and Shanahan [27] refer to polyrhythms with two or more isochronous pulse trains as simple and to polyrhythms with two or more nonisochronous rhythms, whose beats are not harmonically related, as complex. They use the combination of nonisochronous sequences in 2/4 against 6/8 metrical frameworks as an example of a complex polyrhythm. This clear‐cut dichotomous division between simple and complex polyrhythms also contrasts with other studies that conceptualize polyrhythmic complexity as a continuous spectrum (see Section 4.2).

Only one study in our dataset referred to nonharmonically related ratios of isochronous pulse trains (2:3, 3:4, and 4:5) as polymeters [51]. All other studies of our dataset consistently use the term polyrhythm. Only one study directly compares the terms polyrhythm and polymeter. Poudrier and Repp [23] argue that simple polyrhythms with isochronous pulse streams are rarely perceived as having distinct metrical frameworks. However, according to Poudrier and Repp [23], complex polyrhythms with nonisochronous streams afford the perception of two or more distinct metrical frameworks and could therefore be described as polymeters. We recommend avoiding the term polymeter when referring to polyrhythms that consist of two or more nonisochronous or multilayered streams that share a common cycle. In accordance with music education and common practice [14], we propose defining polymeters as the simultaneous presence of two or more meters that maintain their own cycle structures while sharing the same underlying beat [15, 16] (Figure 1D). Although polyrhythms and polymeters can occur in combination, this definition implies that polymeters are neither a special case of polyrhythms nor vice versa.

To avoid confusion in describing beat and meter within polyrhythms we recommend adopting the terminology used in two studies in our dataset. Oshinsky and Handel [39] refer to three‐pulse tappers in a 3:4 polyrhythm as triple meter perceivers and to four‐pulse tappers as quadruple meter perceivers. Focusing on grouping at the subdivision level, Møller et al. [5] refer to the three‐pulse as admitting a quaternary subdivision and the four‐pulse as admitting a ternary subdivision. As outlined in the introduction, both uses of the terms are in line with terminology used in music theory: duple, triple, and quadruple refer to the number of beats per measure (two, three, and four, respectively), whereas binary, ternary, and quaternary refer to the number of subdivisions per beat.

A definition of polyrhythms that we recommend for future use in rhythm perception and production research is “the superposition of two or more nonharmonically related pulses that share a common cycle”; with pulses either being isochronous stimuli, or perceived beats extracted from a nonisochronous or multipart rhythm. Most of the studies in our dataset use nonharmonically related isochronous pulses. Nonharmonically related beats extracted from nonisochronous or multipart rhythms are common in musical practice but remain relatively rare in experimental research. Examples include polyrhythms drawn from commercially available music recordings [12, 27, 52], or the 3:4 polyrhythm used by Stupacher et al. [50]—a 4/4 drum rhythm at 120 BPM, played by hi‐hat, snare, and bass drum, combined with an isochronous woodblock sound at 90 BPM. In most of the reviewed studies, as well as in common musical practice, the superimposed pulses of polyrhythms align at the first beat of a cycle. However, in more advanced artistic practices, musicians may explore aesthetic effects of phase‐shifting the pulses such that their points of alignment occur at temporal locations other than the first beat (when phase shifts follow subdivision IOIs), or the pulses may not coincide at all (when phase shifts do not follow subdivision IOIs). While the first kind of phase‐shift is cognitively accessible and can be performed and perceptually recognized through extensive musical practice, the latter is less likely to be perceived or performed by humans given the constraints of our perceptual and cognitive systems [11]. Therefore, they typically require the mathematical precision available in digital music production [15].

### 4.2 Ratio

The ratio of a polyrhythm defines its temporal structure. It is tightly linked to a polyrhythm's complexity, with ratios such as 2:3 or 3:4 being less complex than ratios such as 5:6 or 7:8. The simplicity of the 2:3 ratio could be one of the reasons why it is used in 34% of the experiments in our dataset. Indeed, eight of the 13 experiments that tested nonmusicians used only the 2:3 ratio, without including any other polyrhythm ratios, likely because it is considered simple enough for nonmusicians to process. Another two experiments with nonmusicians used the 2:3 in combination with other ratios. Importantly, some previous studies used a 2:3 ratio as an ambiguous rhythm without referring to it as polyrhythm in the title, abstract or keywords, and hence did not meet this inclusion criterion for the current dataset (e.g., [18, 19, 20, 24, 53, 54]).

In the reviewed literature, the complexity of polyrhythms has been approached from two angles: objective, mathematical complexity, and subjective complexity, as observed in ratings and behavior. The sum of the ratio is one of the simplest objective measures of polyrhythm complexity, for instance, seven in 2:5 or nine in 4:5 [55]. The higher the ratio sum, the more complex the polyrhythm, as it requires a larger number of events before the pattern repeats, which increases working memory load. Another ratio‐based measure of objective polyrhythm complexity is the lowest common denominator, for instance, 10 in 2:5 or 12 in 3:4. Using this measure, polyrhythm complexity ranks almost the same as it does with the ratio sum: 2:3 is the simplest polyrhythm and ratios like 7:8 or 13:15 are among the most complex. A slight difference between the two measures is that 2:5 and 3:4 are equally complex in the ratio sum, whereas the lowest common denominators would classify 2:5 (10) as less complex than 3:4 (12).

**FIGURE 4:**
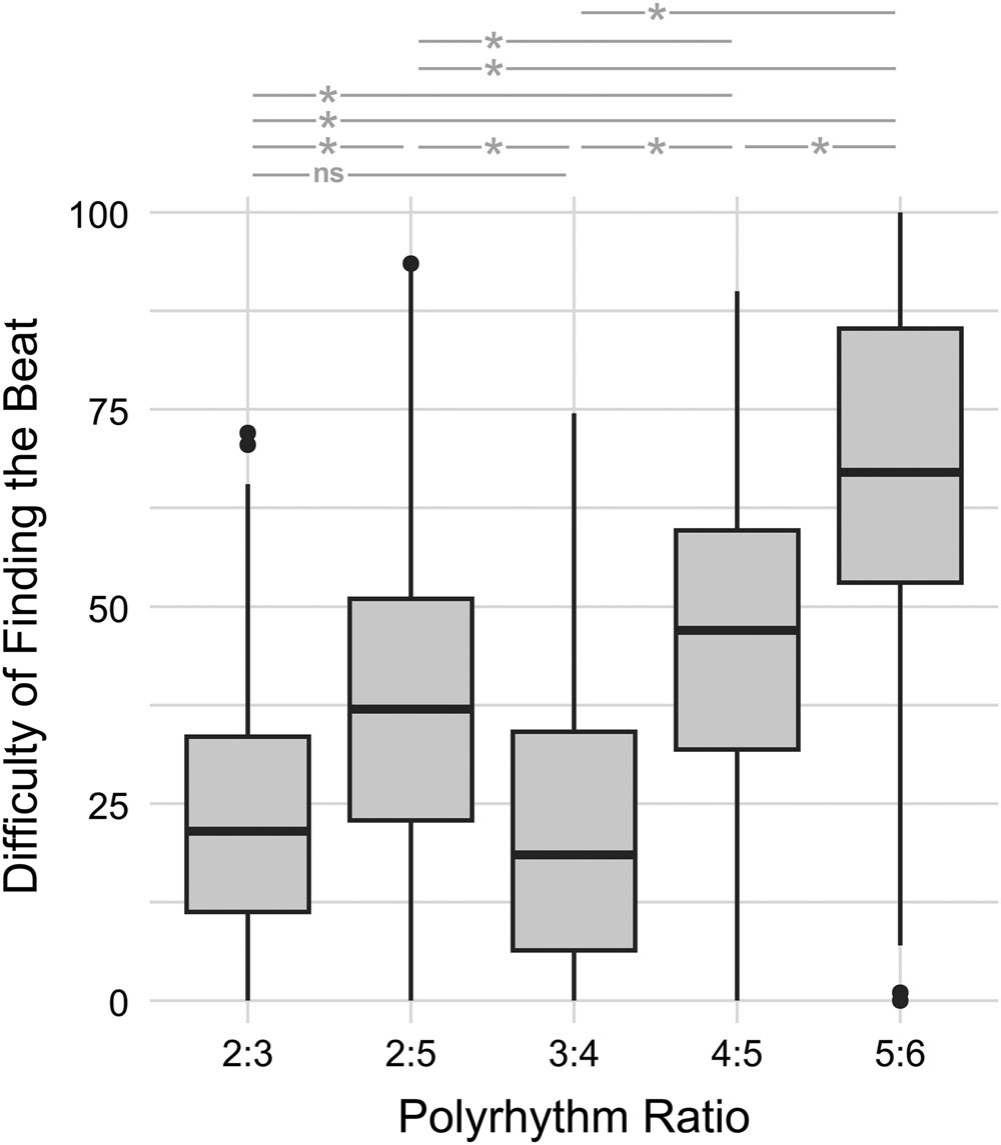
Previously unpublished data from 120 participants in the ratio experiment of Møller et al. [5]. Participants rated the difficulty of finding the beat in five equal‐pitch and equal‐timbre polyrhythms (2:3, 2:5, 3:4, 4:5, 5:6) on a scale from 0 (very easy) to 100 (very difficult). The analysis was based on each participant's mean rating for a given polyrhythm ratio presented with two different subdivision‐IOIs: 125 and 167 ms. For example, a 2:3 polyrhythm with 167 ms subdivision‐IOI has the tempo 180:120 BPM, and a 3:4 polyrhythm with 125 ms subdivision‐IOI has the tempo 120:90 BPM (see Møller et al. [5] and Stupacher et al. [10] for details). An ANOVA indicated a significant effect of ratio (*F*(3.2,384.8) = 157.8, *p* < 0.001). All Tukey‐corrected pairwise comparisons were significant with *p* < 0.01 (marked by *), except for the comparison 2:3 versus 3:4 (ns, *p* = 0.708; cf. Stupacher et al. [10], where 2:3 was perceived as less complex than 3:4).

In polyrhythm perception studies neither the ratio sum nor the lowest denominator measure of complexity appear to fully capture subjective complexity. Recent empirical evidence suggests that 2:5 is perceived as more complex than 3:4 (Figure 4), contradicting both ratio sum and denominator measures. A potential explanation of the differences in perceived complexity between 2:5 and 3:4 polyrhythms is the binary subdivision grouping preference [5, 10], which may interact with effects of musical enculturation and familiarity. In Western listeners, the most common way to perceive 2:5 is with the five pulse as the underlying beat, which affords a binary subdivision grouping (x . x . x . x . x .). Similarly, in a 3:4 polyrhythm, most listeners perceive the binarized three pulse (x . . . x . . . x . . .). The five‐based metrical structure of the 2:5 polyrhythm may be experienced as more complex than the three‐based metrical structure of the 3:4 polyrhythm because Western listeners are generally less familiar with uneven meters such as 5/8 compared to 3/4. Moreover, real‐world, polyphonic music that features polyrhythms usually consists of more complex rhythmic structures than superimposed isochronous pulse trains. Both objective and subjective complexity should therefore not be limited to the ratio alone. Music information retrieval (MIR) features like event density ratio can capture rhythmic variations beyond the relationship between pulses, such as syncopation and microtiming, and are a promising way forward, as demonstrated in a recent study on recordings of musical excerpts [27].

**FIGURE 5:**
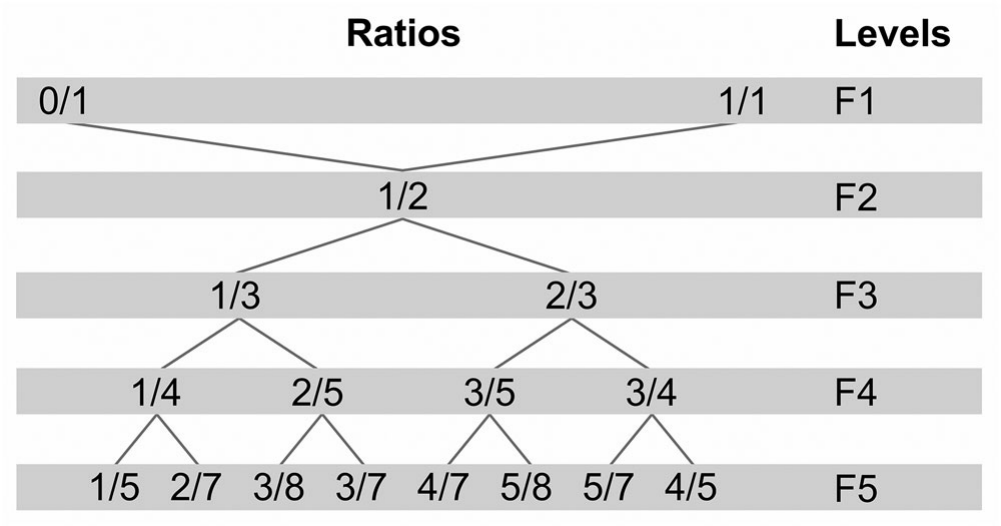
First five levels of the Farey tree.

The Farey tree is an additional ratio‐based measure of complexity, which is frequently applied in the bimanual coordination literature (e.g., [31, 56, 57, 58, 59]). The Farey tree orders ratios from simplest (top) to increasingly complex (lower branches, see Figure 5). This mirrors how movement stability of the rhythmic coordination patterns decreases with increasing sums of numerator plus denominator. The lowest complexity level is 1:1/0:1, then 1:2, branching into the next level with 1:3 and 2:3. On the subsequent level, 1:4 and 2:5 branch from 1:3, and 3:5 and 3:4 branch from 2:3. More complex levels are omitted for brevity. Ratios within the same level of the Farey tree are not necessarily considered equally complex. Instead, the Farey tree is a hierarchical structure modeling behavioral transition points between coordination patterns in which higher levels (such as 2:5 and 3:4) are generally more complex and less stable to produce than lower levels (such as 1:3 and 2:3). Within one branch, more complex ratios commonly transition to simpler ratios, for instance, when increasing movement speed in a bimanual coordination task (e.g., from 3:5 to 2:3 to 1:2) [59].

Apart from the data presented in Figure 4, direct investigations of the subjective complexity of polyrhythm perception and production is scarce. Sensorimotor synchronization and motor coordination tasks are often employed as indirect measures of subjective complexity, with higher accuracy and stability suggesting lower perceived complexity (see Repp [60] and Repp and Su [61] for reviews on sensorimotor synchronization). One example of such measures is the time it takes participants to find a beat and start tapping with polyrhythms, which is shorter for 2:3 than 3:4 and likely related to the fact that 2:3 cycles are shorter than 3:4 cycles [10]. Another example is tapping accuracy, which decreased with increasing polyrhythm ratio sums in Deutsch [62]. Other measures in polyrhythm perception experiments include perturbation detection scores (best in 2:5 and decreasing over 3:5, 4:5, 6:5, and 7:5; Fidali et al. [49]) and brain activity as, for example, measured by fMRI contrasts [52, 63] or differences in the N1 EEG event related potential [64] between iso‐ and polyrhythmic stimuli. In polyrhythm production experiments, indirect measures of complexity include transition to simpler ratios on the Farey tree [57, 58] and movement amplitude which increases with increased complexity [65].

### 4.3 Tempo

The idea that meter perception in polyrhythms depends on tempo was already explored in the earliest publication in our records. Oshinsky and Handel [39] tested whether changes in the tempo of 3:4 polyrhythms affect their perceptual organization. Although they found an effect of tempo on the metrical interpretation of the polyrhythms, their analysis and results are not presented with sufficient detail and transparency to allow for definitive conclusions other than the finding that changes in tempo have some kind of effect on the perceptual organization of polyrhythms. In two later studies, Handel et al. presented various polyrhythms at a wide range of tempi and found a common pattern of tapping behavior: the beat was often perceived as the relatively faster pulse train at slow tempi, as slower pulse train at moderate to fast tempi, and as the cycle at very fast tempi [17, 41]. They relate this pattern to perceptual and motor constraints, suggesting that the underlying beat of a polyrhythm tends to fall within an interval range of 200–800 ms. Moelants and van Noorden [66] largely replicated these findings in 2:5, 3:5, and 4:5 polyrhythms when the pulse trains were separated in pitch by one and five semitones. However, when the pulse trains were separated by a larger interval of 29 semitones, participants tapped less in time with the cycle at faster tempi and more with the slower pulse train, potentially due to improved auditory streaming of the individual pulse trains. Moelants and van Noorden [66] attribute the overall pattern of beat perception to a natural resonance frequency of 2 Hz/500 ms, which aligns well with Handel et al.’s [17, 41] proposed critical window of 200–800 ms.

Møller et al. [5] investigated the role of tempo in polyrhythms, focusing on the structure of subdivision grouping. Using 2:3 and 3:4 polyrhythms with cycle durations between approximately 400 and 5000 ms and equal pitch, they found that participants most commonly tapped in time with pulse trains admitting binary subdivision grouping, that is, the fast three‐pulse train in the 2:3 and the slow three‐pulse train in the 3:4 polyrhythm. At faster tempi, participants predominantly tapped with the cycle, thereby consistently avoiding synchronization with pulse trains admitting ternary subdivision. This finding contrasts with previous studies, but was replicated in a recent study by the same author group, suggesting that the tempo of pulses per se does not predict tapping behavior, whereas the metric structure (binary vs. ternary vs. uneven subdivision grouping) does [10]. Stupacher et al. [10] further showed that metrical interpretations of polyrhythms not only depend on the interaction between tempo and metrical structure of the stimulus, but also on the spontaneous motor tempo (SMT) of participants, that is, the natural rate for repeated, regular movements. In 2:3 polyrhythms, participants with slower SMT tapped at slower rates, however they accomplished this by dividing the binarized pulse train, tapping every second beat of the three‐pulse, instead of tapping every beat of the ternarized two‐pulse. This effect of SMT aligns with Moelants and van Noorden's [66] assumption of a natural resonance frequency, although Moelants and van Noorden acknowledge that their harmonic oscillator approach is a simplification of the body as a complex system.

Using a sensorimotor synchronization task, Handel and Lawson [17] showed that for slow tempi and low‐complexity rhythms like 2:3, most participants tapped the combined rhythmic pattern (i.e., all events of the polyrhythm), which Handel and Lawson refer to as *cross‐rhythm*—also termed *composite rhythm* (see, e.g., [23, 34]). At faster rates, the incidence of tapping the cross‐rhythm dropped rapidly and most participants synchronized with one of the pulse trains. Handel and Lawson [17] additionally showed that for more complex polyrhythms, transitions from cross‐rhythm tapping to isochronous pulse train tapping occurred at slower tempi. In 4:5, for example, 52% of trials were tapped as a cross‐rhythm at the slowest tempo of 80:100 BPM, compared to 81% in the less complex 2:3 at the slowest tempo of 40:60 BPM. One issue here is that Handel and Lawson compare equal cycle durations (i.e., 3 s in the slowest examples of 4:5 and 2:3) and not equal durations of subdivisions. Nonetheless, they concluded that different interpretation strategies, indicated by shifts from cross‐rhythm to pulse train tapping, depend on the tempo and ratio of polyrhythms.

Tempo also plays an important role in polyrhythmic bimanual coordination. Learning to bimanually tap a 3:5 polyrhythm with auditory guidance at a fast tempo (2100 ms cycle) resulted in lower production accuracy of that same polyrhythm than learning with moderate or slow guidance (3000 and 4200 ms cycles, respectively [67]). Findings of Krampe et al. [45] suggest that bimanual performances of polyrhythms at slower tempi are based on integrated timing control across both hands, while performances at faster tempi are better described by parallel timing models that involve more independent control of each hand [45]. Similar findings are reported by Peper et al., who showed that the coupling strength in bimanual performance of a 2:3 polyrhythm decreased as movement frequency increased [68] and that the production of more complex bimanual polyrhythms (here 3:8 and 5:8) becomes less stable with increasing movement frequency, increasing the tendency to shift toward simpler, more stable ratios such as 1:2, 1:3, or 2:3 [58].

### 4.4 Pitch

The relative pitch of polyrhythms’ pulse trains influences their interpretation. In Handel and Lawson [17], participants preferred to tap in time with the lower pitched pulse train, provided that the tempo was comfortable, that is, 200–800 ms intervals between the events of the polyrhythms (see also [41]). The preference for perceiving lower frequencies as the beat was confirmed by Møller et al. [5] in 2:3 and 3:4 polyrhythms and is supported by several studies outside the polyrhythm literature [69, 70, 71]. Beyond the tendency to perceive lower pitches as the underlying beat, pitch separation between the pulse trains in a polyrhythm can influence whether it is perceived as an integrated rhythm or segregated pulses. In nonpolyrhythmic stimuli, larger pitch separation between streams makes perceptual segregation more likely [72, 73], whereas small pitch separations, that is, less than four semitones [73, 74], make it more likely that the two streams are perceived as an integrated rhythm.

Building on these findings from the auditory scene analysis domain [75] several studies modulated the relative pitch of polyrhythms’ pulse trains and used the results to argue whether polyrhythms are perceived as integrated or segregated entities. Some studies showed that smaller pitch separations, which facilitate perceptual integration, also facilitate the production of polyrhythms [76, 77, 78]. Jagacinski et al. [76] used a 2:3 polyrhythm, which, according to Handel and Lawson's [17] findings, is more likely to be perceived as integrated than more complex ratios, and manipulated pitch separation (5 vs. 41 semitones). Jagacinski et al. [76] argued that the better performance with less pitch separation is due to increased perceptual–motor congruence, suggesting polyrhythms are not only perceived, but also performed as integrated patterns.

In contrast, Deutsch [62] designed stimuli with large pitch separation to maximize segregation of polyrhythm streams and compared tapping 1:N ratios as a baseline (i.e., 1:2, 1:3, 1:4, 1:5) to tapping the same tempo in systematically varied (poly)rhythmic contexts (i.e., 2:2, 3:2, 4:2, 5:2; 2:3, 3:3, 4:3, 5:3; 2:4, 3:4, 4:4, 5:4, and 2:5, 3:5, 4:5, 5:5). By keeping the IOIs for the tapped stream constant (e.g., a three‐pulse train that always has an IOI of 400 ms), they assessed tapping accuracy of the same pulse train in different (poly)rhythmic contexts. Deutsch argued that, if the streams can be properly segregated into independent entities, then tapping the same motor tempo should yield similar synchronization accuracies in 1:N and polyrhythm tapping. However, tapping sequences in polyrhythmic contexts consistently resulted in worse motor synchrony across the four tempos (IOIs: 600, 400, 300, 240 ms), indicating that participants perceived integrated patterns, rather than segregated streams in all polyrhythm ratios.

Another strong case for integrated perception of polyrhythms across a range of ratios (2:5, 3:5, 4:5, 6:5, and 7:5) was made by Fidali et al. [49]. When specifically varying pitch separation between polyrhythm pulse trains (up to 20 semitones), they found similar effects of polyrhythm ratio on perturbation detection across levels of pitch separation. Regardless of pitch separation, participants’ detection of perturbations in the target stream (5) was influenced by the other stream (2, 3, 4, 6, or 7), suggesting that perceptual integration, despite being disadvantageous to the task, was unavoidable.

### 4.5 Coordination

Polyrhythms offer a controlled framework to examine the production of temporally complex, bimanual or multilimb patterns. In contrast to polyrhythm perception studies, production studies are less focused on auditory stimulation and also include visual paradigms [44, 79, 80, 81, 82, 83] or even no sensory stimulus [29, 30, 31]. In our dataset, production studies nearly double perception studies, emphasizing the value of polyrhythms for understanding motor constraints, skill acquisition, neural and sensory mechanisms, as well as their applications in clinical rehabilitation and interpersonal coordination.

The specific combination of limbs used can shape both the motor and perceptual organization of the rhythm. Jagacinski et al. [84] showed that the combination of hand and foot is more likely to result in a segregated percept than the combination of two hands. Consistent with this finding, the combination of two hands in the production of polyrhythms is considered a form of integrated motor organization in Summers et al. [78, 85]. A focus on integrated movement patterns has also been suggested to be beneficial when learning to produce a polyrhythm [86, 87]. On the other hand, segregation of the motor patterns (i.e., parallel timing) is more likely to occur with increasing tempo [84], aligning with studies of polyrhythm perception and auditory streaming.

From a neurophysiological perspective, it has been argued that the success of producing complex polyrhythmic coordination may rely on interhemispheric inhibition [88], and that unstable performances of polyrhythms could be related to less effective interhemispheric inhibition [89]. This is based on a model that incorporates four dynamical processes, defined over both motor and premotor cortices, which are coupled through inhibitory and excitatory inter‐ and intrahemispheric connections. Their empirical data suggest that beta frequency oscillations support bilateral phase‐locking. In particular, the model underscores the crucial role of interhemispheric inhibition in reducing the interference between separate movement frequencies during stable motor performance. The timescale of beta synchrony seems to play a key role, as when motor tempo increases, movement‐related desynchronization–synchronization cycles cannot be built up properly, and inhibition may become inadequate, resulting in a reduction of the stability of performance. Therefore, the failure of this interhemispheric inhibition is proposed to lead to behavioral transitions to simpler coordination ratios. Daffertshofer et al. [88] and Houweling et al. [89] argue that their proposed inter‐ and intrahemispheric coupling mechanisms are not restricted to polyrhythmic motor behavior but play a fundamental role in the general stabilization of motor output.

Polyrhythms also provide unique opportunities to study interpersonal coordination. Self‐other integration, a key component of successful interpersonal coordination [90], was found to decrease with increasing polyrhythm complexity [91]. Upon viewing two coordinating figures, participants imagined being one of the figures and indicated that the most self‐other overlap with the other figure was experienced when the two figures were in perfect synchrony. Self‐other merging declined as polyrhythm ratios (2:3, 3:4, and 4:5) increased in complexity and more so for irregular rhythms that contained randomly generated IOIs. Beyond this passive observation, polyrhythms can be created by two individuals coordinating their parts, and auditory polyrhythms can provide external cues for cooperative coordination dynamics that go beyond 1:1 synchrony. However, Heggli et al. [92] showed that even when two participants tapped in 1:1 synchrony, having a nonshared rhythmic context—one participant tapping 500 ms quarter notes in a 120 BPM auditory isochronous sequence and the other one tapping 500 ms half note triplets in a 160 BPM auditory isochronous sequence (i.e., a 3:4 polyrhythm context)—reduced the initial interpersonal synchronization accuracy compared to a shared rhythmic context.

### 4.6 Multimodality

Polyrhythm perception and production are influenced not only by the sensory modalities through which they are presented, but also by the specific combinations of these modalities. Kovacs et al. [80] showed that the difficulty of performing 3:4 and 3:5 polyrhythms can be reduced by providing visual–perceptual information in the form of Lissajous plots. Lissajous feedback is a visualization of the relationship between the two limbs promoting an integrated percept of the movement pattern. Kovacs et al. [81] also showed that 3:4 and 3:5 ratios could be performed remarkably precisely after just 4.5 min of training with Lissajous feedback and without direct visual feedback of the limbs.

Importantly, not all modality combinations are advantageous for polyrhythm production. Lagarde et al. [28] showed that segregating a polyrhythm into distinct perceptual modalities, that is, assigning one pulse train to auditory and the other to tactile input, destabilizes the coordination of 2:3 polyrhythms. Polyrhythm production may therefore benefit more from integrated multimodal perceptual feedback [80, 81] than from distributing different streams across modalities [28]. However, empirical evidence on the benefits and drawbacks of multimodal stimulation and feedback for polyrhythm production accuracy remains scarce.

Many studies on multimodal and cross‐modal rhythm perception in general have concluded that auditory is the dominant modality for temporal accuracy. Therefore, temporal accuracy benefits from adding congruent auditory stimuli to other modalities, but the auditory modality does not gain additional multisensory benefits (for a recent review see; Di Stefano and Spence [93]). It is therefore unsurprising that segregating the streams of a polyrhythm by modality (e.g., substituting an auditory stream with tactile one) leads to less accurate timing compared to auditory‐only presentation, such as in Lagarde et al. [28]. Rhythmic patterns can be recognized in visual, auditory, and tactile modalities, however intersensory composite rhythms in the sense of intersensory Gestalts [94] remain generally unsupported [93]. Therefore, another avenue might be to provide bimodal (e.g., audio‐tactile) stimulation during the motor learning of polyrhythm. Multilimb coordination can then benefit from the haptic signal indicating which limb is playing which event [95].

The benefit of the visual feedback on movement pattern learning in Kovacs et al. [80, 81] might be explained by the fact that the visual feedback consisted of continuous motion rather than discrete visual events co‐occurring with the auditory onsets. Continuous motion is a very appropriate modality for this information [96, 97] due to the high spatial resolution in the visual system [98]. This could explain why continuous integrated feedback can have a benefit for learning the movement patterns, even though the visual system does not have as high of a temporal resolution as the auditory system.

### 4.7 Cultural and Individual Differences

The perception and production of rhythms—including polyrhythms—is closely linked to processes of cultural evolution [99]. Two recent studies in the current dataset, together with a third study published in this special issue, with mostly WEIRD [38] participants, showed a general preference for binary subdivision grouping (e.g., the fast pulse in 2:3 and the slow pulse in 3:4) [5, 10, 100]. Although binarized pulses in music are considered a cultural universal, individuals from certain cultural backgrounds are more likely than Western participants to incorporate ternary and uneven subdivision groupings into their rhythmic priors [101]. Non‐WEIRD participants may therefore show a greater metrical flexibility in the perception and production of polyrhythms.

Our dataset demonstrates a clear link between experimental design choices and musical expertise. Among the 38 experiments with musicians, 34% exclusively used the simple 2:3 ratio, whereas among the 13 experiments with nonmusicians, this proportion nearly doubles to 62%. This pattern suggests that researchers are more inclined to recruit musicians when paradigms include more complex polyrhythms, presumably relying on their stronger sensorimotor synchronization and motor coordination skills. Indeed, musical training has been shown to decrease cycle tapping (which is the simplest form of grouping in polyrhythms) [10], and increase neural tracking when imagining continuations of polyrhythms [50]. Specific motor coordination practice also increases the accuracy in bimanual performances of polyrhythms [82]. However, musicians and nonmusicians use similar perceptual organizations in bimanual performances of polyrhythms [78, 85] and change detection in polyrhythms [32].

Beyond musical training, domain‐specific motor coordination expertise can also shape rhythmic abilities. For instance, elite artistic swimmers, who train to synchronize and coordinate movement to music, are more accurate when producing a 2:3 polyrhythm with hand and foot in time with an auditory input than novice artistic swimmers and nonswimmers [36]. Interestingly, despite the central role of rhythm and motor coordination in dance, systematic investigations of dancers’ polyrhythmic skills remain scarce.

## 5 Conclusion and Recommendations

Given the variability in definitions and operationalizations of polyrhythms, we recommend that future polyrhythm studies provide clear and detailed descriptions of the stimuli used. This includes the ratio, sensory modality, tempo, and, if applicable, amplitude, pitch, and brightness. A definition of polyrhythms that we recommend for future use in rhythm perception and production research is “the superposition of two or more nonharmonically related pulses that share a common cycle”; with pulses either being isochronous stimuli, or perceived beats extracted from a nonisochronous or multipart rhythm. When referring to meter and subdivision grouping in polyrhythms, we recommend using terminology commonly used in music theory: duple, triple, and quadruple refer to the number of beats within one repeating pattern, whereas binary, ternary, and quaternary refer to the number of subdivisions per beat.

As the reviewed literature shows, the subjective complexity of polyrhythms cannot yet be placed on a single continuous scale and has not been systematically tested across perceptual conditions and cultural backgrounds. The heterogeneity of ratio, tempo, pitch, and timbre across the reviewed experiments makes it challenging to compare implicit measures of complexity. In general, the sum ratio provides a good indication of complexity, but as discussed, has its limitations. Importantly, if the sum ratio is to serve as an indicator of the inherent complexity of a polyrhythm, presentation rate should be adjusted for event density. For the same cycle durations, higher sum ratios have higher event densities and will therefore sound more crowded and complex. Comparing different ratios with similar subdivision durations mitigates this problem, although anchoring the subdivision duration may in turn increase complexity for higher sum ratios due to increased working memory demands associated with increased cycle durations. Furthermore, the perceptual complexity of a polyrhythm cannot solely be captured by the mere mathematical relationship between the pulse trains. Therefore, we recommend the use of direct measures of subjective complexity, such as participants’ ratings, in addition to tasks that provide an indirect measure of complexity. These direct measures should be systematically tested across a wide variety of polyrhythms, tempi, perceptual contexts, cultural backgrounds, musical expertise, and dance training.

Several studies suggest that pitch and tempo influence whether polyrhythms are perceived or produced as segregated or integrated patterns. Rather than seeking a general answer about the role of certain stimulus properties, it may be more fruitful to examine under which circumstances polyrhythms are perceived or produced as more integrated or more segregated, and to consider the functions that integration or segregation serve. As with complexity measures, we recommend combining behavioral tasks with subjective and individual measures, as the behavioral studies in our dataset relied heavily on assumptions about how integrated or segregated percepts would manifest in behavior.

Finally, given that most studies in our dataset are based on WEIRD participant samples, future research on polyrhythms should prioritize cross‐cultural investigations and replications—especially of experiments with small sample sizes. The reviewed polyrhythm studies strongly rely on trained musicians, as they are already familiar with the concept of polyrhythm. Therefore, comparisons across the musical expertise spectrum remain scarce. It could be particularly relevant to study the general population who do not have a theoretical understanding of polyrhythmic structure. We also recommend broadening the areas of expertise beyond musicians to include dancers and other groups that train sensorimotor synchronization, such as artistic swimmers [36]. It would be beneficial to model such expertise beyond group comparisons, using continuous variables of music and dance sophistication, that allow inclusion of a broad range of participants from the general population within the same study (Gold‐MSI [102, 103] and Gold‐DSI [104]). Individual differences in the motor domain may further be explored by taking into account SMT [5, 10]. Such approaches can provide a more comprehensive understanding of how culture and expertise shape polyrhythm perception and production, while also informing temporal perception and motor coordination more generally.

The reviewed literature on polyrhythms aligns with the broader rhythm perception literature on several points: auditory dominance exists for polyrhythm perception and production, and polyrhythmic patterns are predominantly perceived, produced, and learned as integrated patterns. These integrated patterns may fall on a spectrum of rhythmic complexity, of which ratio sums and Farey trees are good indications. Considering these similarities, we may expect that findings from polyrhythm perception studies apply to rhythm perception more broadly, and vice versa. Similarly, polyrhythm production fits in a continuum of complex multilimb coordination in the more general motor control literature. Moreover, polyrhythms are an important part of music experiences across cultures and genres and therefore polyrhythm paradigms provide an excellent opportunity to study complex interpersonal coordination in ecological contexts.

## Author Contributions

Patti Nijhuis: conceptualization, methodology, investigation, writing – original draft, writing – review and editing. Cecilie Møller: conceptualization, methodology, investigation, writing – review and editing. Joshua S. Bamford: conceptualization, methodology, investigation. Jan Stupacher: conceptualization, methodology, investigation, formal analysis, writing – original draft, writing – review and editing.

## Conflicts of Interest

The authors declare no conflicts of interest.

## Footnotes

1 In this definition, cycles are perceptual structures. In music notation, these cycles are most commonly separated by bar lines.
